# Substance-P Restores Cellular Activity of ADSC Impaired by Oxidative Stress

**DOI:** 10.3390/antiox9100978

**Published:** 2020-10-12

**Authors:** Jeong Seop Park, Jiyuan Piao, Gabee Park, Hyun Sook Hong

**Affiliations:** 1 Department of Biomedical Science and Technology, Graduate School, Kyung Hee University, Seoul 02447, Korea; godjs@khu.ac.kr; 2 Department of Genetic Engineering, College of Life Science and Graduate School of Biotechnology, Kyung Hee University, Yong In 17104, Korea; fredericpark210@gmail.com (J.P.); yuiop2690@gmail.com (G.P.); 3 East-West Medical Research Institute, Kyung Hee University, Seoul 02447, Korea

**Keywords:** Substance-P, adipose-derived stem cells, oxidative stress, paracrine factors

## Abstract

Oxidative stress induces cellular damage, which accelerates aging and promotes the development of serious illnesses. Adipose-derived stem cells (ADSCs) are novel cellular therapeutic tools and have been applied for tissue regeneration. However, ADSCs from aged and diseased individuals may be affected in vivo by the accumulation of free radicals, which can impair their therapeutic efficacy. Substance-P (SP) is a neuropeptide that is known to rescue stem cells from senescence and inflammatory attack, and this study explored the restorative effect of SP on ADSCs under oxidative stress. ADSCs were transiently exposed to H_2_O_2_, and then treated with SP. H_2_O_2_ treatment decreased ADSC cell viability, proliferation, and cytokine production and this activity was not recovered even after the removal of H_2_O_2_. However, the addition of SP increased cell viability and restored paracrine potential, leading to the accelerated repopulation of ADSCs injured by H_2_O_2_. Furthermore, SP was capable of activating Akt/GSK-3β signaling, which was found to be downregulated following H_2_O_2_ treatment. This might contribute to the restorative effect of SP on injured ADSCs. Collectively, SP can protect ADSCs from oxidant-induced cell damage, possibly by activating Akt/GSK-3β signaling in ADSCs. This study supports the possibility that SP can recover cell activity from oxidative stress-induced dysfunction.

## 1. Introduction

Oxidative stress is an imbalance of free radicals and antioxidants in the body, which can lead to cell and tissue damage. There are several factors that cause oxidative stress and excess free radical production, including obesity, smoking, alcohol consumption, pollution, and chemicals. These risk factors can activate immune cells, which subsequently produce free radicals and impair normal cells. Consistent accumulation of free radicals and cellular damage causes inflammation, contributing to the development of serious diseases including cancer, diabetes, Alzheimer’s, and cardiovascular diseases.

Aging is the progressive loss of tissue and organ function over time. Oxidative stress is believed to be closely related to the aging process because age-associated functional losses are caused by the accumulation of oxidative damage to macromolecules. Although the exact mechanism of oxidative stress-induced aging is unclear, increased free radical production obviously leads to cellular senescence, decreased cell proliferation, and increased inflammatory cytokine production [1]. In addition, several studies have found that with aging, endogenous antioxidant levels, antioxidant enzyme activity, gene expression, and protein levels decrease. This alteration in the antioxidant defense system worsens ROS imbalances and contributes to oxidative-stress-induced aging [1,2,3,4].

Oxidative stress and cellular senescence are involved in several acute and chronic pathological processes such as cardiovascular diseases (CVDs), acute and chronic kidney disease (CKD), neurodegenerative diseases (NDs), macular degeneration (MD), biliary diseases, and cancer. Cardiovascular (CV) risk factors are associated with the inflammatory pathway mediated by interleukin (IL)-1α, IL-6, IL-8, and with increased cellular senescence [5]. The associations between oxidative stress, inflammation, and aging produce a vicious cycle whereby chronic ROS production and inflammation feed each other and accelerate aging and age-related morbidity [6]. Thus, modulating oxidative stress-mediated cellular injury may be a fundamental solution to prevent the progression of lethal diseases in aged individuals.

Stem cells facilitate tissue repair in vivo, and many, such as bone marrow, adipose-derived, and umbilical cord blood stem cells are currently being used as novel treatments for various diseases. The application of mesenchymal stem/stromal cells (MSCs) in regenerative medicine has been intensively studied in many clinical trials, as these cells represent a promising source of multipotent adult stem cells for cell therapy and tissue engineering [7,8,9,10,11,12]. The therapeutic effects of MSCs are generally mediated by various secreted cytokines, growth factors, extracellular matrix proteins, and factors involved in matrix remodeling, as well as different types of extracellular vesicles. 

Currently, as alternative of bone marrow stem cells, adipose-derived stem cells (ADSCs) are being applied extensively in the clinic because they can be easily isolated and cause lower donor-site morbidity [12,13,14]. Aging, oxidative stress, and/or inflammation can affect tissue-resident cells as well as circulating cells, which suggests that stem cells may also be influenced by oxidative stress in vivo. Considering that the aged population is the main population to be treated by stem cell therapy, the restoration of the cellular function of stem cells injured by oxidative stress is likely important.

Substance-P (SP) is an endogenous neuropeptide that interacts with neurokinin receptor 1 (NK-1R). SP has been reported to stimulate cell proliferation and prevent apoptosis under inflammatory or oxidative stress by activating the extracellular signal-regulated kinases 1/2 (ERK 1/2) or Akt and by translocating β-catenin to cell nuclei [15,16,17,18]. SP could suppress inflammation to promote tissue repair in severe diseases by modulating the immune cell profile in circulation-associated and lymphoid organs [19,20,21,22]. Moreover, SP can recover the cellular activity of senescent stem cells [23,24] and stimulate stem cell mobilization from the bone marrow to the peripheral blood [17]. 

Considering these functions of SP, we hypothesized that SP would be able to restore stem cells from oxidative stress-induced injury. To explore the potential recovery role of SP in injured stem cells due to oxidative stress, ADSCs were exposed to H_2_O_2_ at various concentrations. Subsequently, SP was added to the damaged ADSCs and the effect of SP was assessed by evaluating cell viability, cell proliferation, paracrine factors, and early signaling molecules.

## 2. Materials and Methods

### 2.1. Materials

SP and phenylmethylsulfonyl fluoride (PMSF) were purchased from Sigma-Aldrich (St. Louis, MO, USA). Penicillin/streptomycin, 0.25% trypsin-EDTA solution, and phosphate-buffered saline (PBS) were provided by Welgene (Daegu, Korea). Fetal bovine serum and alpha-MEM were purchased from Gibco (Grand Island, NY, USA). An anti-GAPDH antibody (Abcam, Cambridge, MA, USA), and anti-WST-1 antibody (Roche; Indianapolis, IN, USA) were used in this study. Cell lysis buffer, anti-Akt antibody, anti-phospho-Akt antibody, anti-GSK-3β antibody, and anti-phospho-GSK-3β antibody were purchased from Cell Signaling Technology (Danvers, MA, USA).

### 2.2. Cell Culture

Healthy adipose tissues were provided by the Kyung Hee University Hospital Institutional Review Board (eight donors, M6/F2; Seoul, Korea; (IRB# 2016-12-022)). All consents were informed. Fat tissue (two fat tissues (1 × 1 cm) from one donor; total 16 tissues) washed twice with PBS, and treated with collagenase (GMP grade, Vivagen, Los Angeles, CA, USA) for 1 h at 37 °C. Red blood cells and debris were removed using a cell strainer (SPL Life, Pocheon, Korea). The vascular fraction was seeded in α-MEM supplemented with 10% FBS at 37 °C with 5% CO_2_. ADSCs were characterized by analyzing the expression of CD29, CD73, CD105, and CD90 using a fluorescence-activated cell sorting (FACS) Calibur flow cytometer and CELLQuest software (Becton Dickinson, San Jose, CA, USA) (Figure S1). 

### 2.3. Hydrogen Peroxide Exposure Procedure and SP Treatment

Cells were seeded in a 96-well plate at a density of 1 × 10^4^ cells/well or in a 6-well plate with a density of 3 × 10^4^ cells/well. These cells were allowed to adhere to the bottom of the well. Twenty-four hours later, different concentrations of H_2_O_2_ (50, 100, 200, 300, and 400 μM) were added to the wells for 2 h and then removed by changing the culture media. After 24 h, SP was added to each well at a final concentration of 100 nM, and this was repeated 24 h later. 

### 2.4. Wst-1 Assay

Ten microliters of water-soluble tetrazolium salt (WST-1; Roche) solution was added to each well at 10% the total volume of the medium, and the 96-well-plate was incubated for 1 h at 37 °C in 5% CO_2_. After incubation, the optical density values were measured at a wavelength of 450 nm using an Enzyme Linked Immunosorbent Assay (ELISA) microplate reader (Molecular Devices, Sunnyvale, CA, USA).

### 2.5. Enzyme Linked Immunosorbent Assay (ELISA)

The total TGF-β1 and VEGF levels in the supernatants were quantified using ELISA kits, according to the manufacturer’s instructions. In brief, standards and samples were added to the wells of anti-TGF-β1 or anti-VEGF antibody-coated 96-well plates and incubated for 2 h at room temperature. After discarding the supernatant, a horseradish peroxidase-conjugated secondary antibody was added to each well and incubated again for 2 h at room temperature. After rinsing with washing solution three times, 100 µL of substrate solution was added, followed by the addition of 100 µL of stop solution. The optical density was measured at 450 nm using an ELISA microplate reader (Molecular Devices, Sunnyvale, CA, USA).

### 2.6. Preparation of Cell Extracts and Western Blot Analysis

Cells were rapidly washed with chilled 1× PBS and lysed with 1× lysis buffer/1 mM phenylmethylsulfonyl fluoride (PMSF) solution. Cells were then scraped and supernatants were collected by centrifugation (Rotor radius: 70 mm) at 12,000 rpm for 10 min at 4 °C. Protein concentrations of lysates were determined using the bicinchoninic acid (BCA) assay (Thermo Fisher, Rockford, IL, USA). Ten micrograms of lysates were denatured and electrophoresed using sodium dodecyl sulfate-polyacrylamide gel electrophoresis (SDS-PAGE) and transferred to a nitrocellulose membrane. After blocking with 5% skim milk, membranes were incubated with primary anti-Akt, anti phospho-Akt, anti-GSK-3β, anti-phospho-GSK-3β, or anti-GAPDH antibodies, followed by an anti-IgG horseradish peroxidase-conjugated secondary antibody. The blots were processed using enhanced chemiluminescence (GE Healthcare, Buckinghamshire, UK).

### 2.7. Statistical Analysis

All data are presented as the mean ± standard deviation (SD) of more than three independent experiments. *p* values of less than 0.05 were considered statistically significant. Statistical analysis of the data was carried out using an unpaired, two-tailed Student’s *t*-test.

## 3. Results

### 3.1. H_2_O_2_ Impairs the Viability and Morphology of ADSCs

**Figure 1:**
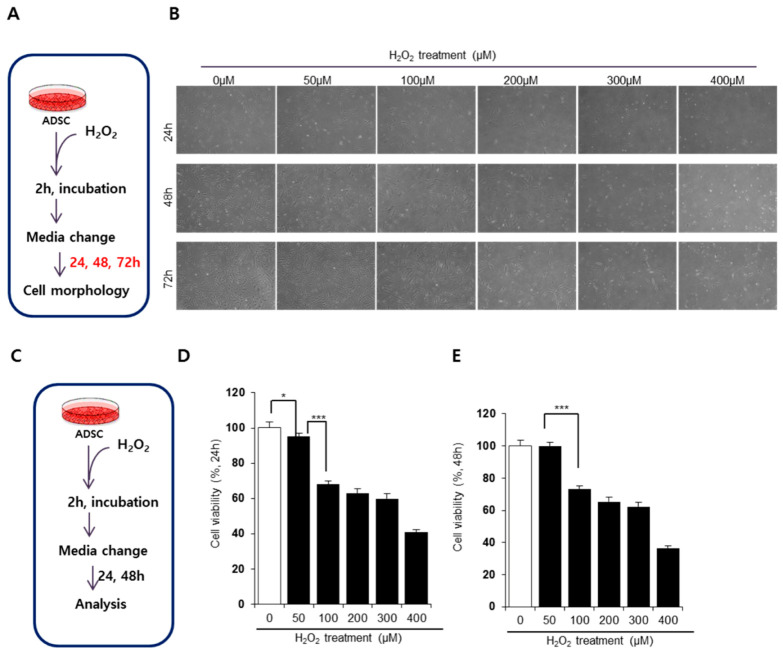
H_2_O_2_ treatment impairs cellular activity of adipose derived stem cells (ADSCs). (**A**,**C**) Experimental scheme for oxidative stress induction in ADSCs. (**B**) Alterations in ADSC cellular morphology depending on the concentration of H_2_O_2_. (**D**–**E**) The viability of ADSCs treated with H_2_O_2_ at 24 h (**D**) and 48 h (**E**) after the removal of H_2_O_2_. The untreated control was set as 100%, and cell viability was expressed as the percentage relative to the activity of the control group. Values of *p* < 0.05 were interpreted as statistically significant (* *p* < 0.05, *** *p* < 0.001). The data are expressed as the mean ± SD of three independent experiments.

Previous reports have induced oxidative stress in vitro by treating cells with H_2_O_2_ for 24 h [25,26]. However, ADSCs appear to be particularly susceptible to H_2_O_2_-dependent oxidative stress, with 1 or 2 h of exposure being sufficient to impair ADSC activity [27,28]. Thus, in this study, we induced oxidative stress in ADSCs by exposing these cells to different concentrations of H_2_O_2_ for 2 h (Figure 1A).

The transient treatment of ADSCs with H_2_O_2_ primarily affected cell proliferation and morphology (Figure 1B). At 24 h after the removal of H_2_O_2_, cell density was significantly different between nontreated and H_2_O_2_-treated conditions. While nontreated cells started to proliferate and became confluent at 72 h, H_2_O_2_-treated ADSCs displayed suppressed cell proliferation. Notably, a distinct difference in cellular density and shape was observed at H_2_O_2_ concentrations above 100 μM. High concentrations of H_2_O_2_ (i.e., 200, 300, and 400 μM) severely impaired the cellular activity of ADSCs, significantly reducing density at 72 h. H_2_O_2_ at 50 μM seemed to damage ADSCs, but these cells appeared to recover as time passed. To compare cellular impairment quantitatively, we determined cell viability at 24 and 48 h after oxidative stress (Figure 1C). H_2_O_2_ treatment decreased cell viability in a dose-dependent manner (Figure 1D,E). Consistent with our results on cell shape and density, ADSCs treated with 50 μM H_2_O_2_ spontaneously restored their activity within 48 h (Figure 1E). However, concentrations above 100 μM H_2_O_2_ reduced cell viability and this did not return to normal levels. Based on these data, we determined that 100 μM H_2_O_2_ induced substantial oxidative damage in ADSCs in vitro. This indicates that transient H_2_O_2_ treatment impairs the cellular viability of ADSCs, which might affect cell survival and the function of ADSCs.

### 3.2. Substance-P Restores Cell Viability of ADSCs Injured by Oxidative Stress

**Figure 2:**
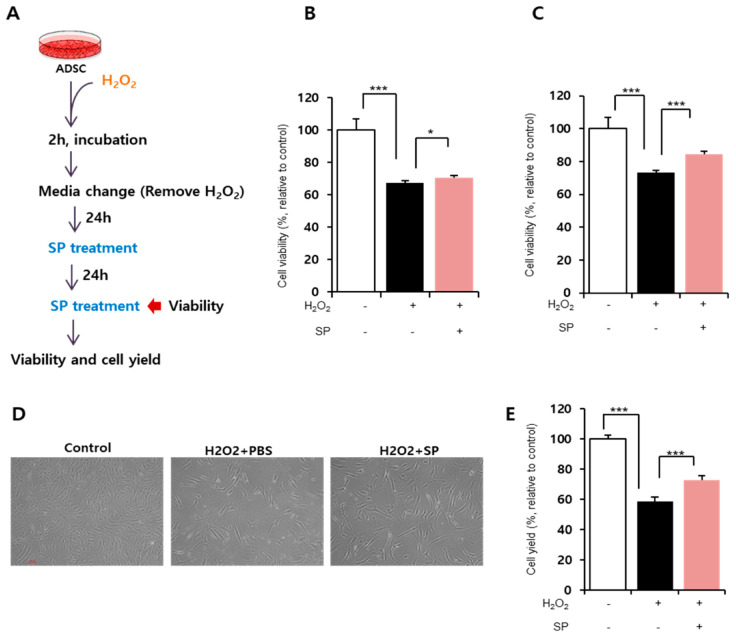
Substance-P improves the viability of ADSCs damaged by oxidative stress. (**A**) Experimental scheme for inducing oxidative stress in ADSCs and the subsequent SP treatment. (**B**,**C**) Cell viability was measured by WST assay at 24 (**B**) and 48 h (**C**) after SP treatment. The untreated control was set as 100%, and cell viability was expressed as the percentage relative to the activity of the control group. (**D**) The representative image of cellular morphology. (**E**) Final cell yield was measured by counting total cell number. Values of *p* < 0.05 were interpreted as statistically significant (* *p* < 0.05, *** *p* < 0.001). The data are expressed as the mean ± SD of three independent experiments.

In order to determine the effect of SP on damaged ADSCs, cells were treated with 100 μM H_2_O_2_ for 2 h and then provided with fresh media. After 24 h, SP was added to the damaged ADSCs at a concentration of 100 nM (Figure 2A). This dose of SP was determined based on previous reports [15,16,17].

Substance-P treatment started to improve ADSC viability within 24 h (Figure 2B; H_2_O_2_ treated: 67.47 ± 1.29%, H_2_O_2_ + SP-treated: 70.4 ± 1.4%, *p* < 0.05) and fully restored cell viability at 48 h (Figure 2C; H_2_O_2_ treated: 73.16 ± 1.83%, H_2_O_2_ + SP-treated: 84.7 ± 3.6%, *p* < 0.001). Cell viability is directly related to cell repopulation. Therefore, we examined the cell yield by counting the total number of cells and comparing it with that of the control. The analysis of cell yield confirmed the restorative effect of SP on damaged ADSCs (Figure 2E, H_2_O_2_ treated: 58.79 ± 1.96%, H_2_O_2_ + SP-treated: 72.97 ± 4.9%, *p* < 0.001, relative to control). SP treatment did not influence cell morphology, but enhanced cell proliferation (Figure 2D).

This revealed that SP treatment enhances the viability of ADSCs impaired by oxidative stress. This effect of SP finally led to the reduced cellular senescence of ADSCs (Figure S2).

### 3.3. Effect of SP on Paracrine Potential of ADSCs Exposed to Oxidative Stress

**Figure 3:**
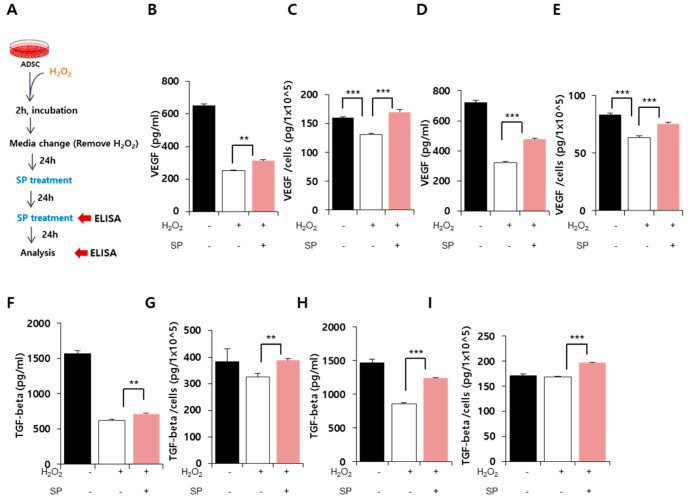
Substance-P recovers the paracrine potential of oxidatively damaged ADSCs. (**A**) Experimental scheme for ADSC with oxidative stress and SP treatment. (**B**–**E**) VEGF in the conditioned medium of ADSCs was quantified using ELISA at 24 (**B**,**C**) and 48 h (**D**,**E**) after the first SP treatment. The absolute concentration of VEGF (**B**,**D**) and secreted amount per 1 × 10^5^ ADSCs (**C**,**E**) were evaluated. (**F**–**I**) TGF-β in the conditioned medium of ADSCs was quantified by ELISA at 24 h (**F**,**G**) and 48 h (**H**,**I**) after the first SP treatment. The absolute concentration of TGF-β (**F**,**H**) and the secreted amount per 1 × 10^5^ ADSCs (**G**,**I**) were evaluated. Values of *p* < 0.05 were interpreted as statistically significant (** *p* < 0.01, *** *p* < 0.001). The data are expressed as the mean ± SD of three independent experiments.

Oxidative stress is well known to decrease cell viability and negatively affect cell function. Stem cells exert their function via paracrine factors, and thus, it is critical to evaluate ADSC cytokine production when investigating the effects of oxidative stress (Figure 3A). VEGF and TGF-β are constitutively produced from MSCs, and their levels are typically reduced by aging or cellular damage [29]. Therefore, VEGF and TGF-β were selected as surrogate markers to represent the paracrine action of ADSCs in this experiment.

VEGF levels from ADSCs significantly decreased after oxidative stress (Figure 3B, 48 h after oxidative stress, Control: 651.8 ± 8.7 pg/mL, H_2_O_2_ treated: 251.4 ± 4.7 pg/mL), and this reduction was sustained by 72 h after oxidative stress (Figure 3D; 72 h after oxidative stress, Control: 721.8 ± 12.9 pg/mL, H_2_O_2_ treated: 321.74 ± 9.77 pg/mL). This indicates that VEGF secretion did not increase significantly between 48 and 72 h, and that impairment of cytokine secretion occurred early. However, SP treatment elevated VEGF production in ADSCs (Figure 3B; 48 h after oxidative stress, H_2_O_2_ + SP-treated: 312.6 ± 8.1 pg/mL; Figure 3D; 72 h after oxidative stress, H_2_O_2_ + SP-treated: 477 ± 5.4 pg/mL).

In this experiment, oxidative stress decreased the total cell number (Figure 2), and thus, it could be inferred that the reduction in cytokine secretion was due to the low cell number. To clarify this, the amount of cytokines was assessed per cell. It was found that oxidative stress disabled the paracrine function of ADSCs (Figure 3C,E). Interestingly, SP treatment increased the concentration of VEGF in the conditioned medium of ADSCs, which might be attributed to their improved ability to produce VEGF as well as the increased cell number. This phenomenon was also observed for TGF-β secretion (Figure 3F,H), with TGF-β levels decreasing following oxidative stress and then recovering after SP treatment (Figure 3G,I).

This suggests that SP is able to improve the paracrine action of ADSCs under oxidative stress, and that repeated SP treatment can intensify the restorative function of SP in damaged ADSCs.

### 3.4. SP Activates Akt Signaling in ADSCs Injured by Oxidative Stress

**Figure 4:**
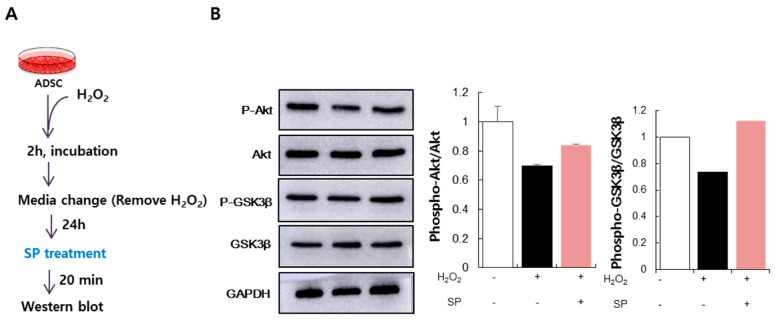
(**A**) Experimental scheme for ADSC oxidative stress and SP treatment. (**B**) Level of phospho-Akt (**B**) and phospho-GSK-3β as detected by Western blotting. Phospho-Akt and phospho-GSK-3β protein expression levels relative to total Akt and GSK-3β were quantified using the Image J program. Expression levels were represented relative to that of the untreated control. The data are expressed as the mean ± SD of three independent experiments.

Typically, when cells are under oxidative stress, signaling associated with cell survival is activated, allowing the cells to survive. The phosphoinositide 3-kinase (PI3K)-Akt pathway is a pro-survival pathway regulated by ROS. When oxidative stress is exerted on cells, Akt is phosphorylated in a PI3K-dependent manner, which induces the phosphorylation and subsequent inactivation of pro-apoptotic factors, including glycogen synthase kinase (GSK)-3 [30,31]. To examine whether the increase in ADSCs viability by SP was accompanied by the activation of Akt signaling, we determined the phosphorylation state of Akt and GSK-3β following ADSCs treatment with H_2_O_2_ and then with SP for 20 min (Figure 4A). ADSCs treated with H_2_O_2_ failed to maintain phosphorylated Akt levels, whereas SP treatment promoted Akt phosphorylation (Figure 4B). Additionally, GSK-3β, a downstream effector of Akt signaling and a pro-apoptotic molecule [14], was phosphorylated and inactivated following SP treatment. The expression levels of phospho-Akt and phospho-GSK-3β were quantified relative to the levels of total Akt and GSK-3β (Figure 4B). Taken together, these results demonstrate that SP can activate Akt/GSK-3β signaling, which contributes to the SP-induced recovery of oxidatively damaged ADSCs.

## 4. Discussion

Aging and oxidative stress are highly associated with inflammation and the development of mortal diseases [1,21]. To combat oxidative stress-related diseases, antioxidants are typically applied from natural compounds or various medicines; however, their effects were equivocal, and are often accompanied by unwanted side effects. Therefore, additional treatment options with increased efficacy are urgently needed.

Most studies deal with oxidative stress to study retinal disease [6,16]. Stem cell therapies have emerged as an exciting option in the treatment of a variety of diseases including those that are related to oxidative stress. ADSCs are among the most popular stem cells to be used in novel therapies [12,32], as they have a high repopulation potential and their tissue of origin (adipose) is easily accessible. Ideally, therapies involving ADSCs would involve their autologous transplantation; however, ADSCs from diseased or aged patients may not be fully functional given the heightened levels of free radicals and inflammation in the individual. Indeed, we found that ADSCs from diseased animals have low repopulation rates and decreased cytokine secretion with a lack of differentiation potential even in early passages [33]. Moreover, a recent study corroborated the impairment of ADSCs by oxidative stress. Several studies have also indicated that, compared to other cells, stem cells are more susceptible to damage due to free radicals [27]. Thus, the effect of oxidative stress on stem cells should be taken into consideration for the application of stem cell therapy and endogenous regeneration.

In this study, oxidative stress was induced by treating ADSCs with H_2_O_2_ for 2 h. We found that this transient exposure to H_2_O_2_ was sufficient to affect ADSC activity and function, but was not completely detrimental to the ADSC population. H_2_O_2_ treatment altered cell morphology, reduced cell viability, inhibited the proliferation of ADSCs, and decreased their paracrine potential. This impairment could not be restored by removing the oxidant, which might lead to cellular senescence and death.

In an attempt to rescue ADSCs from oxidative stress, SP was employed. SP treatment of injured ADSCs enhanced cell viability and restored the paracrine potential of ADSCs. Moreover, at an early time point, SP activated the Akt/Gsk-3β pathway, which was downregulated by oxidative stress, and might contribute to the improvement of cell survival. Differentiation potential was also improved by SP but its effect was so slight, comparing to that of cell viability and cytokine secretion. Therefore, it was inferred that SP can augment cell survival and secretome production (rather than stemness), which might contribute to enhanced differentiation potential, to some extent. Notably, paracrine factors including VEGF and TGF-beta are deeply involved in osteogenesis [34,35,36]. SP could restore VEGF and TGF-beta production from ADSC with H_2_O_2_. This might suggest the possibility for the correlation between paracrine potential and osteogenesis (Figure S3)

In conclusion, this study demonstrated that SP could stimulate the recovery of ADSCs under oxidative stress, possibly by promoting cell proliferation through the activation of Akt/GSK-3β signaling. SP is anticipated to enhance the activity of ADSCs from aged or diseased individuals. Constant treatment of SP is anticipated to further augment the restoration of impaired stem cells.

## Supplementary Materials

The following are available online at https://www.mdpi.com/2076-3921/9/10/978/s1, Figure S1: The analysis for marker expression of ADSC; Figure S2: Beta-galatoxidase staining of ADSC with H_2_O_2_ and SP; Figure S3: The differentiation ability of ADSC with H_2_O_2_ and SP.
