# Supplementary material for: Substance-P Restores Cellular Activity of ADSC Impaired by Oxidative Stress
Source: Antioxidants (Basel). 2020 Oct 12;9(10):978. doi: 10.3390/antiox9100978 (PMC7601553; doi:10.3390/antiox9100978)
Supplement: Supplementary file 1 [file antioxidants-09-00978-s001.pdf]

Supplementary Figure 1

Marker	positive (%)
CD105	99.54
CD73	99.53
CD34	0.54
CD44	99.71
CD29	99.5
CD45	0.02

Supplementary Figure 1. The analysis for marker expression of ADSC
ADSC was treated with FITC or PE-conjugated antibody against CD105, CD73, CD34,CD44,CD29 and CD45 for 5 min and then analyzed by FACS (Novocyte)

Supplementary Figure 2

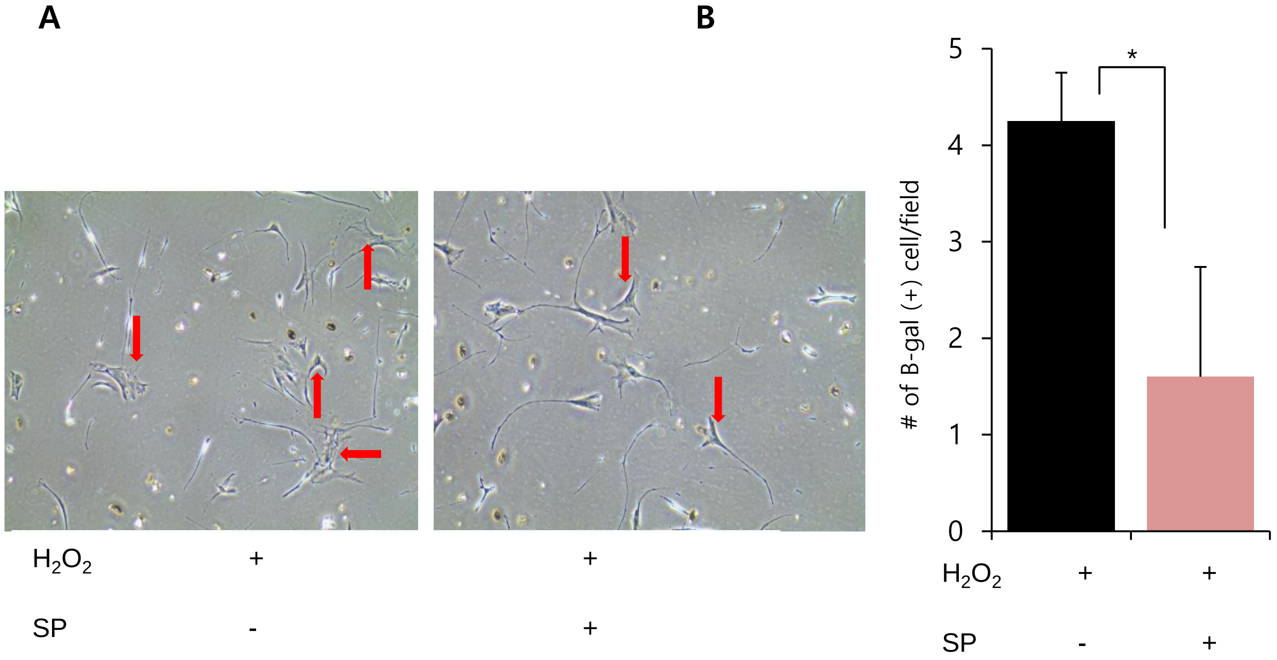

Supplementary Figure 2. Beta-galatoxidase staining of ADSC with H_2O_2 and SP
(A) The representative image of beta-gal staining at 72h after SP treaatment. ADSC was treated with H_2O_2 for 2h and then, H_2O_2 was removed by providing fresh media. 24h later, SP was added. These cells were cultured for 24, 48 and 72h post the removal of H_2O_2. Cells were fixed with formalin and beta-galatoxidase (+) cells were stained by cellular senescence staining kit. Red arrow: beta-gal stained cells (B) beta-galatoxidase (+) cells were counted in same filed at 96h after H_2O_2 stimulation. Values of $p < 0.05$ were interpreted as statistically significant (*$p < 0.01$). The data are expressed as the mean ± SD

Supplementary Figure 3

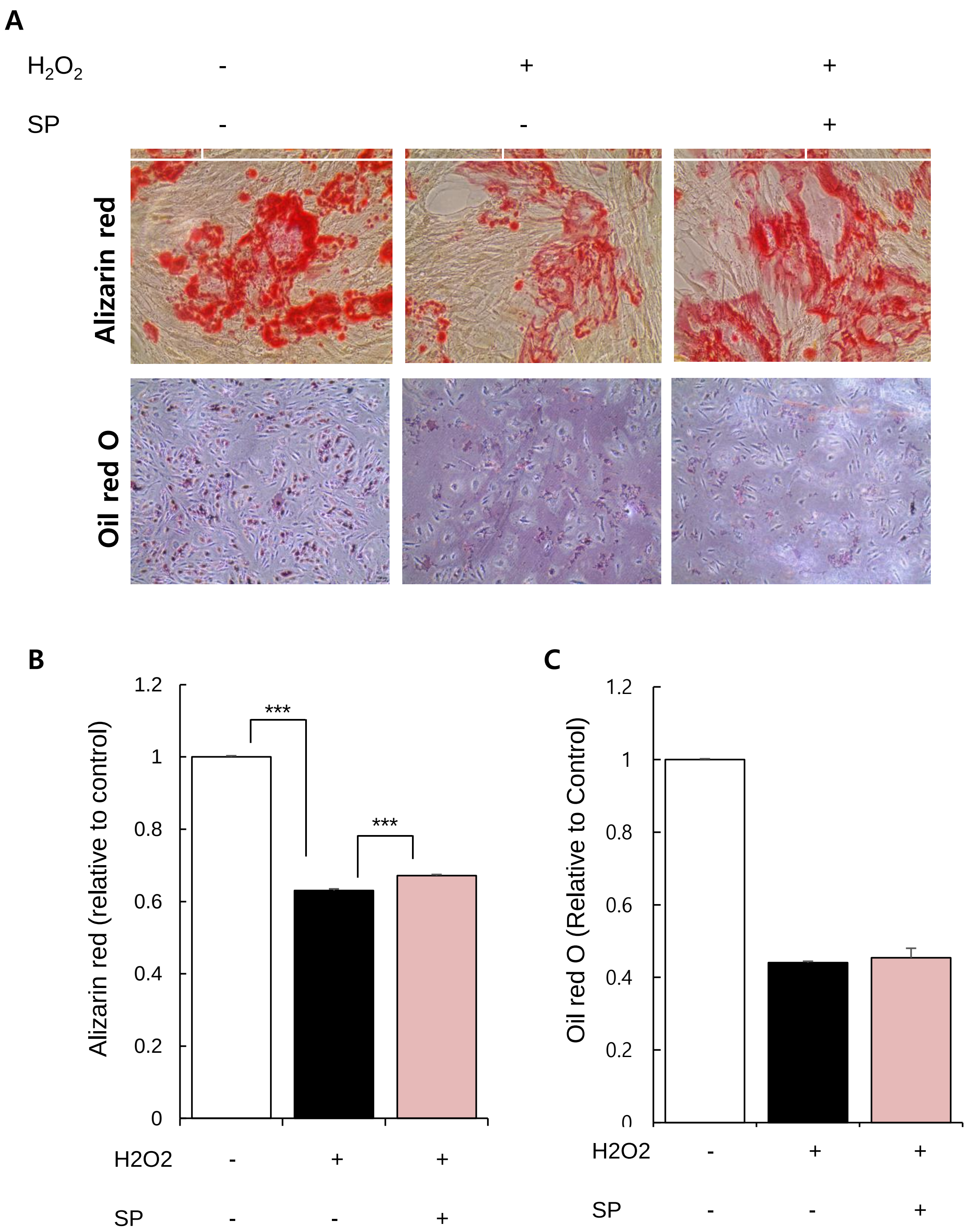

Supplementary Figure 3. The differentiation ability of ADSC with H_2O_2 and SP
(A) ADSC was treated with H2O2 and then, SP was added. Theses cells were induced into osteoblast and adipocyte for 20 days. Calcium deposition for osteogenesis was determined by alizarin red staining and lipid droplet was visualized by oil red o staining. (B-C) Quantification of alizarin red and oil red O was performed by extracting the stained and measuring optical density (560nm for alizarin red; 490nm for oil red o). Values of $p < 0.05$ were interpreted as statistically significant (***$p < 0.001$). The data are expressed as the mean ± SD of three independent experiments.
